# RSA-PT: A Point Transformer-Based Semantic Segmentation Network for Uninterrupted Operation in a Distribution Network Scene

**DOI:** 10.3390/s25082370

**Published:** 2025-04-09

**Authors:** Deyu Nie, Linong Wang, Shaocheng Wu, Zhenyang Chen, Yongwen Li, Bin Song

**Affiliations:** 1 Engineering Research Center of Ministry of Education for Lightning Protection and Grounding Technology, School of Electrical Engineering and Automation, Wuhan University, Wuhan 430072, China; 2024282070146@whu.edu.cn (D.N.); wushaocheng@whu.edu.cn (S.W.); 2022302071014@whu.edu.cn (Z.C.); 2024282070189@whu.edu.cn (Y.L.); 00007609@whu.edu.cn (B.S.); 2 School of Electrical Engineering and Automation, Wuhan University, Wuhan 430072, China

**Keywords:** point cloud, semantic segmentation, distribution network, uninterrupted operation, point transformer

## Abstract

The digitization of uninterrupted operation in the distribution network is of great significance for improving people’s quality of life and promoting economic development. As an important means of achieving digitization, point cloud technology is crucial to the intelligent transformation of distribution network. To this end, the authors embedded the improved RSA (residual spatial attention) module and modified the loss function of network, proposing a deep learning network called RSA-PT for the semantic segmentation of a distribution network scene point cloud. According to the requirements of uninterrupted operation in the distribution network, the authors segmented the point cloud into the following ten classes: high-voltage line, low-voltage line, groundline, tower, ground, road, house, tree, obstacle, and car. Model and attention mechanism comparison experiments, as well as ablation studies, were conducted on the distribution network scene point cloud dataset. The experimental results showed that RSA-PT achieved *mIoU* (mean intersection over union), *mA* (mean accuracy), and *OA* (overall accuracy) indicators of 90.55%, 94.20%, and 97.20%, respectively. Furthermore, the *mIoU* of RSA-PT exceeded the baseline model by 6.63%. Our work could provide a technical foundation for the digital analysis of conditions for uninterrupted operation in distribution networks.

## 1. Introduction

Uninterrupted operation in the distribution network allows for the inspection, maintenance, and fault handling of distribution equipment without power outages. It plays an irreplaceable role in reducing downtime and improving supply reliability [1]. In recent years, digitalization has become an important development direction in the power industry. Smart meters [2] and digital switchgear [3] have enhanced the level of distribution services. However, in the field of uninterrupted operation in the distribution network, the operating environment is highly complex. As a result, manual on-site surveys are required before the operation to assess the working conditions and propose work plans. This process leads to inefficiencies in the operations [4,5].

Currently, point cloud technology is being increasingly applied in digital surveys [6]. We aimed to apply point cloud technology to distribution networks, particularly for the on-site condition survey of uninterrupted operation. This could improve work efficiency and enhance the stability of distribution network operations. In the transmission field, Xu et al. [7] extracted conductors from a point cloud to conduct potential threat analysis, ensuring the safety of power transmission. In the distribution line, drone technology, which combines UAVs with attitude sensing technology, has also begun to be used for distribution network inspections [8]. These provide a technological foundation and valuable experience for the application of point cloud technology in distribution networks.

Point cloud segmentation is the preliminary study for the digital analysis of conditions for uninterrupted operation in the distribution network using point cloud technology. The distribution network scene is quite complex, with many variables to consider for uninterrupted operation [9]. Different electrical equipment has different safety restrictions, including high-voltage line, low-voltage line, and groundline, as well as tower. Given that uninterrupted operation may involve the use of insulated aerial work platforms, it is necessary to segment the road and other ground features. Additionally, it is essential to segment classes of the point cloud, such as obstacle, car, house, and tree classes, that occupy ground space. The segmentation of these four classes is based on various factors, such as immovability, their status as public facilities, distance restrictions between electrical equipment and residential areas, and their potential hazards. The distribution network scene is complex, with significant disparities in the quantities of various point cloud classes. The similarities in structure between streetlights and utility towers, as well as between road and ground features, further complicate the segmentation of point cloud in the distribution network scene.

Point cloud segmentation techniques can be divided into two classes, namely machine learning and deep learning methods. Machine learning relies heavily on manual experience and expertise, and its performance is often limited on large-scale datasets [10]. In contrast, deep learning methods can automatically learn meaningful features from raw point cloud data, handle large-scale and complex point cloud data, and reduce the dependence on manual feature extraction [11]. The point transformer [12] introduced the advantages of the Transformer into point cloud processing. Through the self-attention mechanism, it could adaptively learn the relationships between points in the point cloud, effectively aggregating semantic information regardless of the distance between points. However, some classes in the distribution network point cloud data have similar structures and are affected by significant noise pollution, an issue that the point transformer has not yet addressed.

Therefore, using the point transformer as the baseline model, we proposed the RSA-PT, an RSA (residual spatial attention)-empowered point transformer for semantic segmentation of the point cloud in the distribution network scene. The main contributions of this paper are as follows:

(1) To address the issues of structural similarity and noise, we embedded the RSA module in order to enhance the method’s ability to extract local features and reduce the impact of class noise during classification and applied it before the encoder.

(2) To address the issues of class imbalance and sample size discrepancies, we proposed a loss function—LogWeight cross-entropy loss—that assigns different weights to different classes based on logarithmic operations and inverse frequencies. This loss function is used in conjunction with the standard cross-entropy function during training.

(3) We proposed the RSA-PT model, which divides the point cloud data of the distribution network scene into four types of electrical equipment, namely tower, highline, lowline, and groundline, and six types of environmental conditions, namely road, ground, tree, house, obstacle, and car. Our model outperforms the baseline model by 6.63%, 5.81%, and 1.93% in the *mIoU*, *mA*, and *OA* indicators, respectively.

## 2. Related Work

Point cloud data, with its ability to accurately represent the geometric shape and spatial position of objects in three-dimensional space, has been widely applied in various fields. Its processing methods have evolved through several stages with the development of technology [13].

### 2.1. Machine Learning

In the early stages, machine learning algorithms were first applied to the field of point cloud processing. Algorithms, such as support vector machines (SVMs) [14] and random forest [15], are used for simple point cloud classification. This approach can achieve certain results when handling point cloud data with relatively simple structures and distinct features. For a complex point cloud in a distribution network scene, where objects have diverse shapes and the spatial layout is complicated, it is difficult to extract comprehensive and discriminative features. This often leads to poor segmentation accuracy, and the feature extraction methods lack generality and adaptability when faced with different scenes [16].

### 2.2. Deep Learning

With computational power advancing rapidly, deep learning has seen wide application across fields. Initially, researchers tried to apply 2D image processing-based CNNs to point cloud data [17]. However, due to the disorder and irregularity of point cloud data, direct CNN application faced major hurdles. Voxelization [18] and 2D projection [19] were proposed to make a point cloud data CNN, yet they caused substantial spatial information loss and segmentation errors.

To directly process unordered point cloud data and to tap its semantic info, specialized deep-learning architectures emerged. PointNet [20] was a pioneer, using symmetric functions and MLPs to handle unordered point cloud sets for classification and segmentation. PointNet++ [21] then improved on it with a hierarchical feature-learning design, enhancing local and global feature extraction.

Later models, like SparseUnet (based on SpConv [22] and the Minkowski [23] Engine), PointCNN [24], DGCNN [25], and CAC [26], boosted segmentation performance on public datasets. Inspired by the Transformer’s success in natural language processing, the point transformer [12] was proposed. Its self-attention mechanism adaptively captures point-to-point relationships in the point cloud, facilitating better semantic aggregation for accurate segmentation.

### 2.3. Research on Distribution Network

Currently, there is almost no research on point cloud segmentation in the distribution network field. In the past, scholars have conducted some studies on the transmission line. For instance, Yu et al. [27] proposed Powerline-Net for the semantic segmentation of the point cloud in an ultra-high-voltage transmission line, while Wang et al. [28] introduced CA-PointNet++ for the semantic segmentation of the point cloud in high-voltage transmission corridors, providing technical support for transmission line inspections. However, the point cloud scene for transmission line is relatively simple, typically consisting of a transmission line, groundline, tower, and vegetation. In contrast, the distribution network scene involves far more classes, with significant variations in point cloud features across different classes. Additionally, the complexity of the distribution network scene leads to more data noise. Han et al. [29] proposed NF-PTv2 for semantic segmentation in distribution network scenarios. However, its segmentation accuracy is relatively low and fails to meet the requirements of uninterrupted operation surveys in distribution networks. Our study addressed these shortcomings and improved the segmentation accuracy.

## 3. Materials and Methods

In this section, we rethink the overall architecture of the point transformer. We also introduce our proposed network design and architecture for semantic segmentation of point cloud in distribution network scene.

### 3.1. Baseline Model

The point transformer is a typical encoder–decoder network that can perform semantic segmentation tasks without the need for auxiliary modules, such as convolutions. The main modules and functions of the network are as follows.

**Positional Encoding.** The point transformer is built on the self-attention mechanism, which is the key foundation for the semantic segmentation of the point cloud. Due to the unordered nature of point cloud data, the network needs to introduce new spatial position information to capture the spatial relationships between points. In the point transformer, trainable parametric positional encoding is introduced. The positional encoding function is shown in the following Equation (1):

(1) $$\delta =\theta (p_{i}-p_{j})$$

where *p*_*i*_ and *p*_*j*_ represent the 3D coordinates of point *i* and point *j*, and the encoding function *θ* is a multi-layer perceptron (MLP) consisting of two linear layers and a ReLU layer.

**Figure 1:**
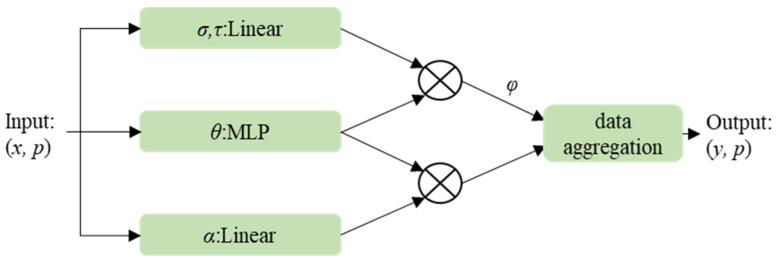
The architecture of the PTL module.

**Point Transformer Block.** The point transformer layer (PTL) module is constructed with the self-attention mechanism at its core. The PTL module serves as the feature aggregation operator and is the central component of the network [12]. Its architecture is shown in the following Equation (2):

(2) $$y_{i}=\sum_{x_{j}\in X\left(i\right)}\alpha \left(\varphi \left(\sigma \left(x_{i}\right)-\tau \left(x_{j}\right)+\delta \right)\right)⊙\left(\rho \left(x_{j}\right)+\delta \right)$$

where *Χ*(*i*) ⊆ *Χ* represents the local neighborhood of point *x_i_*, defined by the KNN (K-nearest neighbor). Specifically, for each point *x_i_*, the KNN method is employed to identify its K-nearest neighboring points, which collectively form the local neighborhood *Χ*(*i*). Subsequently, the features of these neighboring points are aggregated using the self-attention mechanism to generate the output feature *y_i_* for point *x_i_*. Here, *σ*, *τ*, and *ρ* are feature transformations, φ is the mapping function, and *α* is the normalization function. The architecture of the PTL module is shown in Figure 1. 

Based on the PTL module, the core module of the model, namely the point transformer block (PT Block), is constructed. This module plays a key role in feature processing and optimization. It takes the feature vectors *x*, which include relevant 3D coordinate information *p* as input, performs feature aggregation on the input point features, and generates new feature vectors for each data point, providing more enriched and expressive feature data for the subsequent network.

**TransitionDown.** In the main architecture of the model, it is necessary to reduce the number of points and aggregate relevant features of the point cloud. The TransitionDown module identifies a point set *P*_2_ with important features from the input point set *P*_1_ using farthest point sampling, and then aggregates the features of *P*_1_ into *P*_2_ through KNN.

**TransitionUp.** The model uses an encoder–decoder architecture, with the decoder implemented through the TransitionUp module. It maps the input point set *P*_2_, which has been downsampled by TransitionDown, to a superset *P*_1_ where *P*_1_ ⊃ *P*_2_. To achieve this, the features of each point in the input point set are linearly processed, and then trilinear interpolation is used to map the features to the higher-resolution point set *P*_1_. The features processed by trilinear interpolation are then fused with the features provided by the encoder through skip connections.

**Figure 2:**
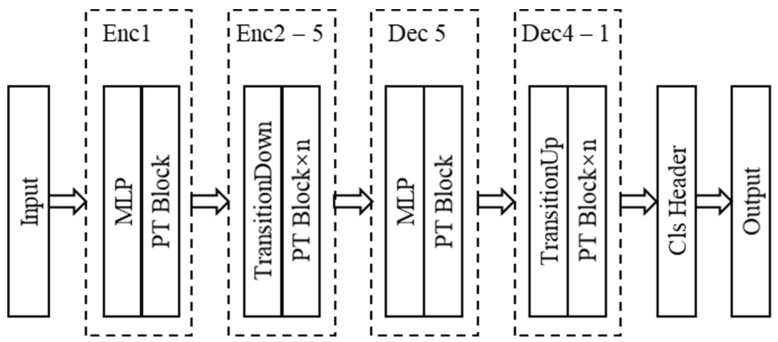
The architecture of the point transformer.

**Network Architecture.** The point transformer network is entirely based on point transformer layers, point transformations, and pooling to achieve point cloud semantic segmentation, with a U-Net architecture model constructed around the point transformer block [12]. The model’s feature encoder and decoder consist of five stages. The encoder progressively downsamples the point set, with the sampling rates of each stage being [1, 4, 4, 4, 4]. After each stage, the number of points in the set becomes [N, N/4, N/16, N/64, N/256], where N is the number of points in the input point set. The architecture of the point transformer is shown in Figure 2.

A detailed introduction to the model can be found in [12].

### 3.2. Optimization Design

When considering applying the point transformer to semantic segmentation in a distribution network scene, we analyzed the characteristics of the s3dis (Stanford 3D Indoor Spaces) point cloud dataset [30] and the point cloud dataset for the distribution network. Compared to the s3dis dataset, our distribution network point cloud dataset is more complex, with more interference and a larger disparity in the number of points for each class. Directly applying the point transformer to the distribution network segmentation resulted in poor performance, especially for the obstacle and car classes. In RSA-PT, we made improvements to the model.

#### 3.2.1. Residual Spatial Attention (RSA) Module

The point transformer uses the self-attention mechanism to enhance feature extraction, focusing on global long-distance features. However, in the distribution network scene, there are similar structures in the tower and obstacle classes, such as streetlights, which are column-like and spatially adjacent. The structures of the ground and road features are also similar, making them prone to segmentation errors. Additionally, due to the complexity of the point cloud in the distribution network scene, there is a significant amount of noise from such classes as the distribution equipment, environment, and obstacle. Therefore, we aimed to design an attention mechanism that can achieve denoising effects and focus on local features.

**Figure 3:**
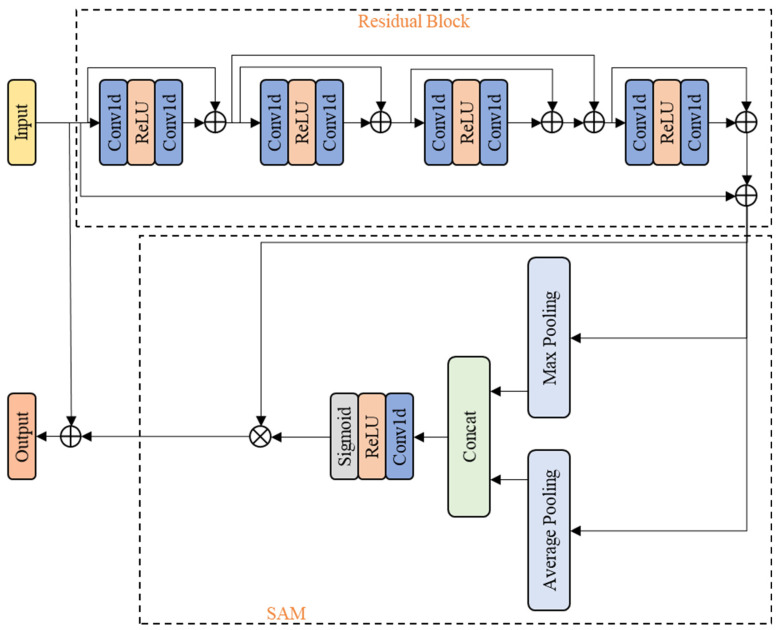
The architecture of the RSA module.

The design of the RSA module is based on [31], which integrates the spatial attention mechanism with other neural network functional layers to focus on useful information and filter out non-informative features. Considering the structural differences between the point cloud data and the originally applicable image data, we replaced some of the neural network functional layers in the module to suit the semantic segmentation of the point cloud in the distribution network scene. In [31], the attention module consists of a residual block and the spatial attention module (SAM). The residual block contains multiple convolutional layers and ReLU. The architecture of the RSA module is shown in Figure 3.

The residual block of RSA contains four RES (residual calculation) layers, each consisting of Conv1d-ReLU-Conv1d architecture. These RES layers extract rich local features from the point cloud through multiple skip connections between the layers. After the residual block, we add SAM to complement the extraction and learning of global feature information. SAM concatenates the results of the max pooling and average pooling along the channel dimension, compresses the input, and then applies Conv1d, ReLU, and sigmoid to weight the features. Our RSA module includes multiple skip connections, which allow feature extraction and fusion between different RES layers, further enhancing the ability to extract point cloud features. Multi-level feature fusion is achieved through skip connections, reducing the loss of feature information. Meanwhile, the original spatial information is supplemented for SAM, which focuses on key spatial regions. The detailed process of RSA data handling is as follows.

The processing method of the RES layer for the input point cloud is shown in the following Equation (3):

(3) $$y_{i}=res(x_{i})=\mathrm{Conv}1d(\mathrm{ReLU}(\mathrm{Conv}1d(x_{i}))$$

The processing method of the residual block [31] for the input point cloud is shown in the following Equation (4):

(4) $$x_{4\text{-}1}=(x+((x+(x+y_{4})+y_{3})+y_{2})+y_{1})$$

where *x* ∈ *ℝ^b^^×c^^×n^*, *b* is the batch size, *c* is the number of channels, and *n* is the number of points in the point cloud. *x_i_* is the input of the *i*-th RES layer. *y_i_* is the output of the *i*-th RES layer.

*x*_4-1_ is passed as the input to SAM, which includes two operations, namely channel pooling and convolution. Channel pooling consists of max pooling and average pooling. The pooled results are concatenated along the feature dimension to obtain the tensor *x_compress_*. Then, *x_compress_* is convolved to obtain *x_out_*, and weighted using a sigmoid function. The computation method is shown in the following Equations (5) and (6):

(5) $$x_{out}=\mathrm{ReLU}(\mathrm{Conv}1d(x_{compress}))$$

(6) $$s=\frac{1}{1+\exp(-x_{out})}$$

The weighted result is used with the residual block result, and the final skip connection is performed to obtain the output *x*_5-1_ of the RSA module. The computation method is shown in the following Equations (7) and (8):

(7) $$x_{5}=x_{4\text{-}1}\times s$$

(8) $$x_{5\text{-}1}=x+x_{5}$$

The overall mathematical logic of the RSA module [31] is shown in the following Equation (9):

(9) $$x_{5\text{-}1}=s\times (x+((x+(x+y_{4})+y_{3})+y_{2})+y_{1})+x$$

#### 3.2.2. LogWeight Cross-Entropy Loss

Our baseline model used the standard cross-entropy loss function to measure the difference between the model’s predictions and the true labels. While it plays a positive role to some extent, when applied to point cloud segmentation in the distribution network scene, the disparity in the number of points across classes becomes significant. The standard cross-entropy loss function did not account for the imbalance in sample size across classes, leading the model to favor accurate predictions for classes with a larger number of samples, while neglecting classes with fewer samples. This results in a negative impact on the overall classification performance.

Therefore, we added LogWeight cross-entropy loss on top of the standard cross-entropy loss. This loss dynamically calculates the weights for each class based on the number of point clouds and uses logarithmic operations and inverse frequency to place greater emphasis on the classes with fewer point clouds, thus mitigating the impact of class imbalance.

The computation of the standard cross-entropy loss function is shown in the following Equation (10):

(10) $$CE(y,p)=-\sum_{c=1}^{10}y_{c}\log(p(y=c\left|x\right.)$$

The computation of the LogWeight cross-entropy loss function is shown in Equations (11)–(13):

(11) $$\omega_{c}=\frac{1}{\log(n_{c}+1e-10)}$$

(12) $${\hat{\omega }}_{c}=\omega_{c}/\sum_{c=1}^{10}\omega_{c}$$

(13) $$LWCE(y,p)=\sum_{c=1}^{10}{\hat{\omega }}_{c}(-y_{c}\log(p(y=c\left|x\right.))$$

where *y_c_* is the one-hot representation of the true class label (i.e., if the true class is *c*, then *y_c_* = 1; otherwise, *y_c_* = 0), and *p*(*y* = *c*|*x*) is the predicted probability distribution. *ω_c_* is the weight for each class, and ${\hat{\omega }}_{c}$ is the normalized weight for each class. Among them, *c* takes values from 1 to 10, which refers to the 10 classes of point clouds segmented in the article.

### 3.3. RSA-PT Architecture

**Figure 4:**
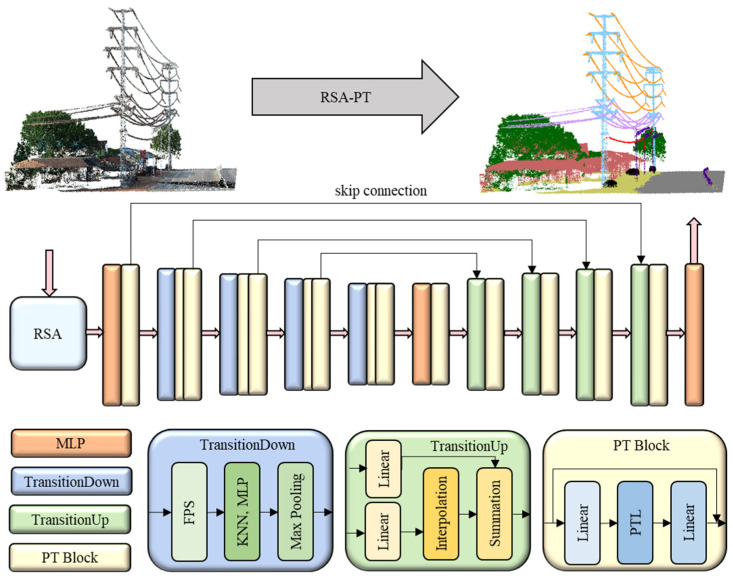
The architecture of the RSA-PT.

The architecture of the RSA-PT model is shown in Figure 4. After processing through RSA, the input passes through a five-layer encoder and decoder network, and finally outputs the segmentation result via the classification head. The number of PT Blocks used in the encoding layers of our model is [1, 2, 2, 2, 2].

## 4. Experiment

### 4.1. Dataset

The distribution network point cloud dataset was obtained using a drone equipped with a LiDAR system. The type of drone used was the DJI M300 RTK (DJI Innovations, Shenzhen, China), and the LiDAR system was the Livox L1 (Livox Technology Co., Ltd., Shenzhen, China). The acquired point cloud data include 45 distribution network scenes. Based on the requirements for uninterrupted operation in the distribution network, we manually segmented the acquired point cloud data into four types of electrical equipment point clouds, namely the tower point cloud, high-voltage line (highline) point cloud, low-voltage line (lowline) point cloud, and groundline point cloud. Additionally, the data were divided into six types of environmental condition point clouds, namely the road point cloud, ground point cloud, tree point cloud, house point cloud, obstacle point cloud, and car point cloud. The point cloud data were divided into six regions, which were used as the training set, testing set, and validation set, respectively. The number of points for each class is shown in Table 1, where “average” represents the number of point clouds for each class divided by the number of scenes.

### 4.2. Experimental Conditions

In this section, we outline the conditions under which the experiments were conducted, including hardware and software configurations, as well as the experimental setup for the point cloud segmentation tasks in the distribution network scene.

The experimental environment of our model is shown in Table 2. The optimizer used in the RSA-PT is AdamW. The remaining experimental parameters are set as shown in Table 3.

### 4.3. Indicators

In the model evaluation, we used overall accuracy (*OA*), mean accuracy (*mA*), intersection over union (*IoU*), and mean intersection over union (*mIoU*) to assess the segmentation performance. The calculation methods for these four indicators [28] are shown in the following Equations (14)–(17):

(14) $$OA=\frac{TP+TN}{TP+TN+FP+FN}$$

(15) $$mA=\sum_{c=1}^{10}OA_{c}/10$$

(16) $$IoU=\frac{TP}{TP+FP+FN}$$

(17) $$mIoU=\sum_{c=1}^{10}IoU_{c}/10$$

where *OA_c_* is the *OA* for the *c*-th class of point cloud, and *IoU_c_* is the *IoU* for the *c*-th class of point cloud. *TP* is when both the ground truth and prediction are positive; *TN* is when both are negative; *FP* is when the ground truth is negative but the prediction is positive; *FN* is when the ground truth is positive but the prediction is negative. These metrics help evaluate the model’s performance by comparing predictions with actual labels.

### 4.4. Results

Table 4 shows the indicator results for the point cloud segmentation. Compared to the baseline model, the proposed RSA-PT improved the indicators *mIoU*, *mA*, and *OA* by 6.63%, 5.81%, and 1.93%, respectively. It showed improvements in most classes, with the only exception being the highline class, where the *IoU* indicator was 0.31% lower than that of the baseline model. For classes with structurally similar characteristics, such as tower, ground, and road, the *IoU* indicators increased by 6.16%, 5.03%, and 9.86%, respectively. The class with fewer samples, i.e., the groundline class, also improved by 5.59%. In contrast, for the classes with the poorest segmentation performance in the baseline model, namely obstacle and car, the segmentation performance of RSA-PT improved by 17.22% and 23.07%, respectively. The *mIoU* indicator is the most commonly used indicator for evaluating model segmentation performance. We conducted experimental comparisons between RSA-PT and three other models, namely two SparseUnet models (Minkunet and Spunet, which used the Minkowski Engine and SpConv, respectively; they were shown in [32]) and the baseline model point transformer. RSA-PT outperformed Minkunet, Spunet, and the point transformer by 8.34%, 8.17%, and 6.63%, respectively, in terms of the *mIoU* indicator.

In Minkunet and Spunet, there were classes with outstanding segmentation performance. The house class in Minkunet and the tower and car classes in Spunet achieved the highest results among several models. However, the class imbalance was quite severe. The segmentation performance of certain classes such, as lowline, ground, and obstacle, in the former, and ground and obstacle in the latter, was poor and did not meet the requirements for the analysis of uninterrupted operation in distribution network. The *IoU* indicators for the first eight classes segmented by RSA-PT were all above 90%, and the segmentation performance of the last two classes also met the requirements for uninterrupted operation in the distribution network.

**Figure 5:**
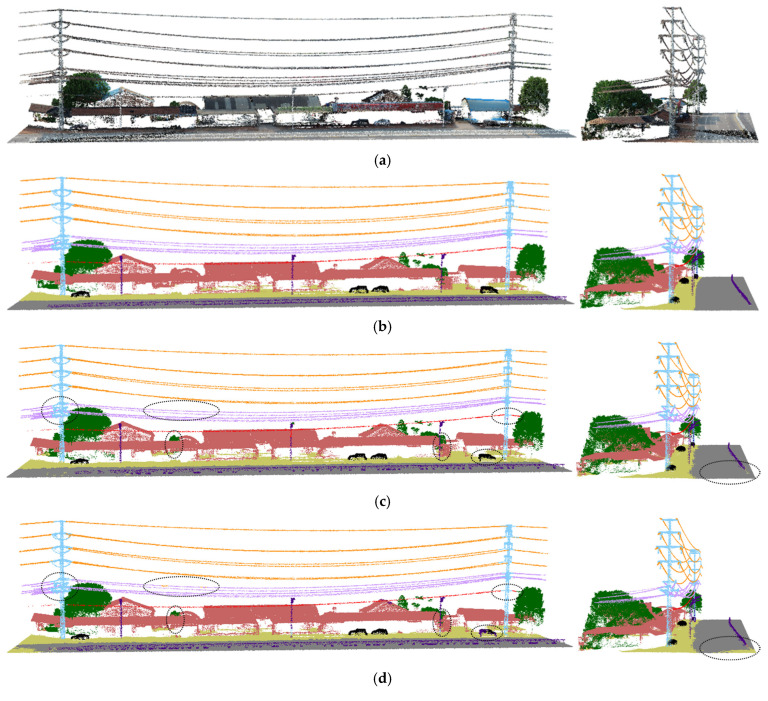

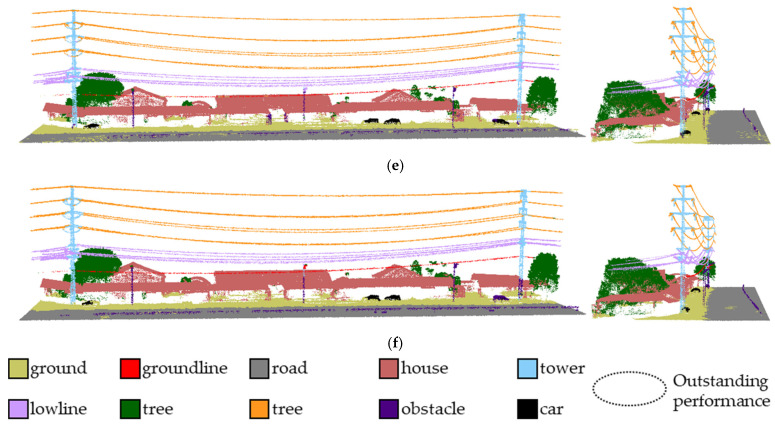
Segmentation results for Scene 1 in the validation set (viewed from two angles). (**a**) Original image, (**b**) Ground truth, (**c**) RSA-PT (ours), (**d**) Point transformer (baseline), (**e**) Spunet, (**f**) Minkunet.

**Figure 6:**
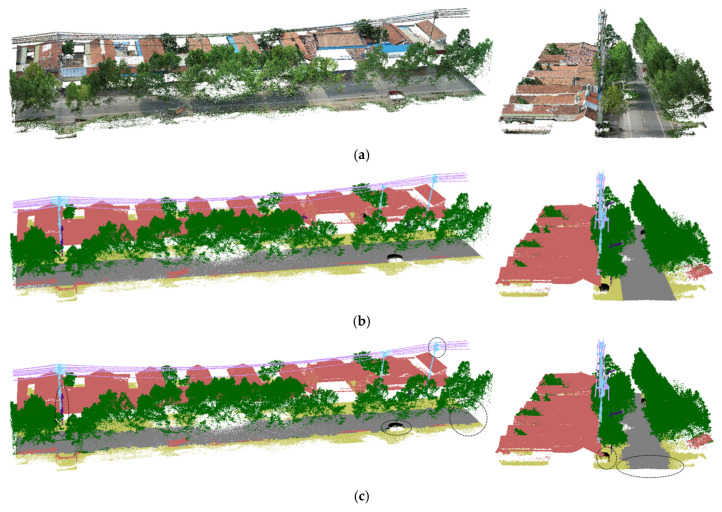

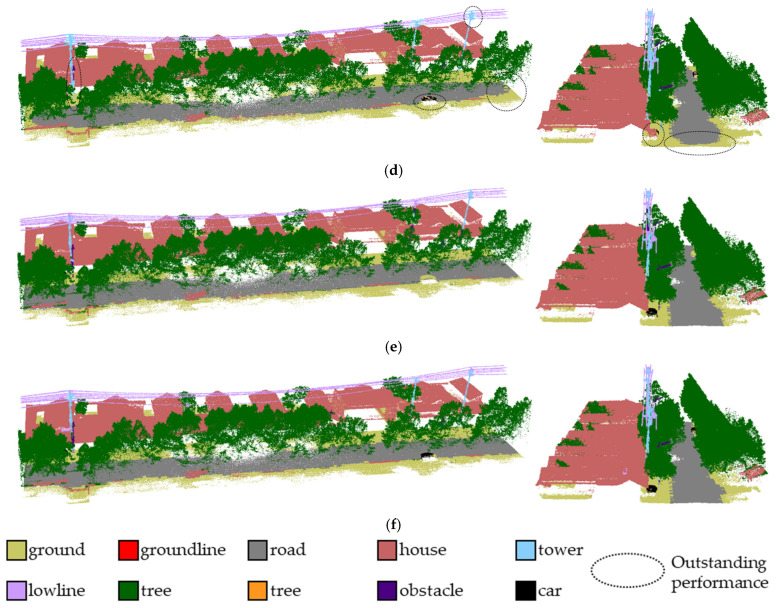
Segmentation results for Scene 2 in the validation set (viewed from two angles). (**a**) Original image, (**b**) Ground truth, (**c**) RSA-PT (ours), (**d**) Point transformer (baseline), (**e**) Spunet, (**f**) Minkunet.

Figure 5 and Figure 6 show the segmentation results taken from the validation set. In both the open scene and the scene with multiple trees, RSA-PT demonstrated improved segmentation performance on electrical equipment. In the baseline model, the boundary segmentation between road and ground was poor, and some streetlights in the obstacle class were misclassified as towers. Additionally, the classification performance of car was not ideal, while our model addressed this issue to some extent. Compared to the other two SparseUnet models, RSA-PT showed better overall segmentation performance, with superior segmentation results in most classes.

Specifically, in the segmentation result images of Spunet and Minkunet, many point clouds were misclassified as ground, with frequent misclassifications of obstacle, car, and other classes. This has a significant impact on the analysis of conditions for uninterrupted operation in distribution network. Additionally, both models misclassified the structure connecting tower and conductor as a line, which greatly affects the safety analysis of uninterrupted operation in the distribution network. In contrast, RSA-PT effectively addressed these issues, validating the effectiveness of RSA-PT.

## 5. Discussion

In this chapter, we validated the role of the RSA module and the LogWeight cross-entropy loss. The results in this section are all obtained through the statistical analysis of the results of five experiments.

### 5.1. Ablation Experiment of a Single Module

Table 5 and Table 6 show the segmentation performance indicators under individual optimizations. As can be seen from the table, the use of the RSA module and the addition of the loss function improved the *mIoU* indicator by 4.82% and 4.22%, respectively. When only the RSA module was used, the *IoU* indicators for ground, road, and tower classes showed improvements, and the *IoU* indicators for obstacle and car showed significant improvements. The *IoU* indicators for obstacle and car classes showed significant improvements, increasing by 19.76% and 16.56%, respectively. These classes are precisely those that have similar structures and a relatively large amount of noise. When exclusively integrating the LogWeight cross-entropy loss, significant improvements were observed in the segmentation metrics for small-sample point cloud classes, specifically groundline (*IoU* + 4.05%), obstacle (*IoU* + 14.18%), and car (*IoU* + 14.34%).

When only the RSA module was used, the *IoU* indicators for the tower, ground, road, obstacle, and car classes showed significant improvements. The purpose of using the RSA module was to distinguish between classes with similar structures and to reduce the impact of noise. The improvements for the tower and obstacle classes, as well as ground and road, came from the differentiation of similar structures, while obstacle and car were classes in the distribution network point cloud with more noise. The improvements in these five classes validate the effectiveness of the RSA module.

When only the LogWeight cross-entropy loss was added, the *IoU* indicators for groundline, ground, road, obstacle, and car classes showed significant improvements. The purpose of this loss function was to address class imbalance and increase the weight of low-sample classes. The results show that classes with low sample sizes, such as groundline, obstacle, and car, had notable improvements. Meanwhile, ground and road, the classes with higher sample sizes, also showed considerable improvements. The reason for this may be the improved feature extraction for low-sample classes, which in turn enhanced the model’s ability to extract global features. This fully validates the effectiveness of LogWeight cross-entropy loss.

### 5.2. Attention Mechanism Comparison Experiment

In this section, we embed attention mechanisms at the same position in the baseline model to validate the effectiveness of the RSA module. The attention mechanisms we embed include the channel attention (CA), spatial attention (SA), convolutional block attention module (CBAM), and squeeze-and-excitation attention (SE) mechanism.

The results in Table 7 show that when attention mechanisms were embedded in the baseline model, our RSA outperformed CA, SA, CBAM, and SE by 2.61%, 2.36%, 3.47%, and 4.12%, respectively, in terms of the *mIoU* indicator for point cloud segmentation. Notably, under the influence of the other four attention mechanisms, the *IoU* for some classes showed noticeable improvement, but this was achieved at the cost of segmenting more classes less effectively, exacerbating the class imbalance in segmentation. RSA, however, performed better than the other attention mechanisms in this regard.

Among the other four attention mechanisms, CA, CBAM, and SE showed exceptionally poor segmentation performance for certain classes, which was detrimental to the analysis of uninterrupted operation in the distribution network. When using SA, the segmentation performance was 2.43% lower than RSA in terms of *mIoU*, with much better results for the road class. However, SA sacrificed many electrical equipment segmentation results, which could have impacted the accuracy of the safety analysis for uninterrupted operation in the distribution network. The results demonstrated that the Residual Block and SAM in the RSA module, along with its skip connections, were meaningful for improving the network’s feature extraction capability.

### 5.3. RSA Kernel Size Comparison Experiment

In the RSA module, feature extraction is performed using multiple Conv1d layers, and the size of the convolutional kernel will affect the overall segmentation performance of the network. Therefore, in this section, we will validate whether the kernel size used in the model is suitable for point cloud segmentation in distribution network scene.

The results in Table 8 show that the kernel size of 3 provided the best performance for point cloud segmentation in the distribution network scene. The *mIoU* indicator was at least 3.3% higher than that of other kernel sizes.

The 1 × 1 convolution kernel does not extract spatial information but is generally used to process feature information within channels. The 3 × 3 convolution kernel, on the other hand, can extract local feature information. In the RSA module, multiple convolution blocks use 3 × 3 convolution kernels to gradually expand the receptive field, enhancing sensitivity to information at different scales. As the convolution kernel size increases, the receptive field also expands. However, the larger the convolution kernel, the lower its sensitivity to local features, giving more emphasis to global features. Nevertheless, the point transformer excels at capturing global features through its self-attention mechanism. Therefore, the 3 × 3 convolution kernel is more suitable for point cloud segmentation in the context of uninterrupted operation in the distribution network.

## 6. Conclusions

The semantic segmentation of point clouds in a distribution network scene is a crucial technology for the digital survey of field conditions in uninterrupted operation. It significantly improves the work efficiency and safety of such operations. Distribution network point clouds are relatively complex, and to achieve effective segmentation, we proposed the optimization of the RSA and the LogWeight cross-entropy loss. Based on these improvements, we introduced the RSA-PT deep learning point cloud segmentation network, which segmented the point cloud into the following ten classes: highline, lowline, groundline, tower, ground, road, house, tree, obstacle, and car.

We conducted semantic segmentation experiments on point clouds in a distribution network scene. Compared to our baseline model, i.e., the point transformer, we achieved a 6.63% improvement in the *mIoU* indicator. Additionally, we compared RSA-PT with other deep learning point cloud algorithms. The results on the validation set show that RSA-PT outperforms other models in terms of *mIoU*, *mA*, and *OA* indicators. For the ten classes, the IoU for most classes is also the best among all models.

We performed ablation experiments on RSA-PT, and the results demonstrate that the two proposed improvements significantly enhance the model’s segmentation performance. Both improvements notably boost the segmentation of classes, such as obstacle and car, which are difficult for the baseline model to handle. Moreover, there are significant improvements in the segmentation of road and ground boundaries as well as in the accuracy of small-sample classes. Furthermore, we determined the optimal kernel parameters for the RSA module, which led to better segmentation results in distribution network scene.

The results indicated that RSA-PT outperforms existing models in the semantic segmentation of a distribution network point cloud, with a 6.63% improvement in the overall *mIoU* indicator compared to the point transformer. However, the segmentation accuracy for complex structures, such as the obstacle point cloud, still requires improvement. We divided the point clouds in the uninterrupted operation of the distribution network scene into ten classes, which provides a technical foundation for the digital analysis of uninterrupted operation conditions and lays the groundwork for applying point cloud technology to the distribution network. In the future, we will continue to focus on research into the semantic segmentation of point clouds with complex structures.

## Figures and Tables

**Table 1:** The number of points for each class in the dataset.

Dataset	The Number of Points									
	Highline	Lowline	Groundline	Tower	Ground	Road	House	Tree	Obstacle	Car
All	251,492	887,017	24,140	748,639	19,296,511	10,154,856	8,912,154	19,616,119	632,100	450,739
All (average)	13,658	19,711	1609	16,636	428,811	225,663	212,194	445,820	19,753	13,658
Validation	53,901	142,491	4735	129,317	2,724,724	2,082,916	2,269,804	2,534,364	37,703	62,646
Validation (average)	26,950	20,355	1578	18,473	389,246	297,559	378,300	362,052	6283	10,441

**Table 2:** Experimental environment configuration.

Configuration	Version
CPU	12th Gen Intel(R) core (TM) i5-12400F
GPU	NVIDIA GeForce RTX 4080
Operating system	Linux = 6.8
Python	3.8.19
CUDA	11.7

**Table 3:** Experimental parameters.

Parameter	Value
Grid size	0.6
Kernel size of RSA	3
Batch size	2
Epoch	300
Learning rate	0.006
Decay weight	0.05

**Table 4:** Segmentation performance of each model.

Model	*IoU*										*mIoU*	*mA*	*OA*
	Highline	Lowline	Groundline	Tower	Ground	Road	House	Tree	Obstacle	Car			
Minkunet	89.10	78.76	86.87	86.84	76.10	86.35	**97.70**	88.03	45.40	76.98	82.21	90.82	95.78
Spunet	89.96	87.89	88.53	**96.53**	68.10	82.91	97.36	87.36	42.89	**82.32**	82.38	89.25	95.95
Point Transformer	**96.72**	95.26	88.30	86.94	87.18	83.24	96.77	98.15	50.48	56.16	83.92	88.39	95.27
RSA-PT (ours)	96.41	**96.01**	**93.89**	93.10	**92.21**	**93.10**	97.38	**98.17**	**67.70**	79.23	**90.55**	**94.20**	**97.20**

The units for *IoU*, *mA*, and *OA* are percentages. Bold values indicate the optimal values.

**Table 5:** Results of the single module ablation experiment.

Module	*IoU*										*mIoU*	*mA*	*OA*
	Highline	Lowline	Groundline	Tower	Ground	Road	House	Tree	Obstacle	Car			
Point Transformer	96.58 ± 0.25	95.06 ± 0.29	88.37 ± 1.34	86.63 ± 1.45	86.84 ± 1.16	83.14 ± 1.40	96.62 ± 0.25	98.02 ±0.19	50.27 ± 1.43	56.02 ± 2.06	83.41 ± 1.71	88.39 ± 1.68	95.15 ± 0.82
+RSA	**97.31 ± 0.64**	93.81 ± 0.68	86.47 ± 1.42	92.17 ± 1.16	88.30 ± 1.44	85.43 ± 1.59	96.39 ± 0.58	97.80 ±0.44	**70.03 ± 1.95**	72.58 ± 1.72	88.23 ± 1.39	91.77 ± 1.58	95.70 ± 0.66
+Loss	90.65 ± 0.63	94.56 ± 0.77	92.42 ± 1.09	87.56 ± 1.52	91.43 ± 1.07	90.54 ± 1.34	96.82 ± 0.68	97.74 ±0.57	64.45 ± 1.59	70.36 ± 1.53	87.63 ± 1.30	92.18 ± 1.22	96.62 ± 0.70
RSA-PT (ours)	96.47 ± 0.38	**96.16 ± 0.38**	**93.75 ± 0.50**	**93.08 ± 0.56**	**92.16 ± 0.68**	**93.24 ± 0.52**	**97.39 ± 0.46**	**98.08 ± 0.34**	67.54 ± 1.24	**79.17 ± 1.31**	**90.05 ± 0.82**	**94.18 ± 0.56**	**97.11 ± 0.48**

Bold values indicate the optimal values.

**Table 6:** Comparative table of module contributions.

Module	*IoU*										*mIoU*	*mA*	*OA*
	Highline	Lowline	Groundline	Tower	Ground	Road	House	Tree	Obstacle	Car			
RSA	**0.73**	−1.25	−1.90	5.54	1.46	2.29	−0.23	−0.22	**19.76**	16.56	4.82	3.38	0.55
Loss	−5.93	−0.5	4.05	0.93	4.59	7.40	0.20	−0.28	14.18	14.34	4.22	3.79	1.47
RSA-PT	−0.11	**1.10**	**5.38**	**6.45**	**5.32**	**10.10**	**0.77**	**0.06**	17.27	**23.15**	**6.64**	**5.79**	**1.96**

Bold values indicate the optimal values.

**Table 7:** Results of the attention mechanism comparison experiment.

Attention	*IoU*										*mIoU*	*mA*	*OA*
	Highline	Lowline	Groundline	Tower	Ground	Road	House	Tree	Obstacle	Car			
Base	96.58 ± 0.25	**95.06 ± 0.29**	88.37 ± 1.34	86.63 ± 1.45	86.84 ± 1.16	83.14 ± 1.40	96.62 ± 0.25	**98.02 ± 0.19**	50.27 ± 1.43	56.02 ± 2.06	83.41 ± 1.71	88.39 ± 1.68	95.15 ± 0.82
CA	89.86 ± 0.92	69.70 ± 1.35	88.64 ± 1.23	95.54 ± 0.98	**92.30 ± 0.89**	94.30 ± 0.85	**97.64 ± 0.58**	96.66 ± 0.72	65.31 ± 1.45	65.81 ± 1.56	85.62 ± 1.42	88.97 ± 1.23	96.45 ± 0.49
SA	86.68 ± 0.95	91.82 ± 0.60	83.41 ± 1.15	92.84 ± 0.72	89.82 ± 0.92	93.36 ± 0.80	97.48 ± 0.48	90.54 ± 0.68	54.78 ± 1.07	71.12 ± 1.23	85.87 ± 0.92	91.65 ± 0.72	96.05 ± 0.40
CBAM	89.07 ± 0.55	88.68 ± 0.85	86.48 ± 0.92	**96.54 ± 0.34**	86.15 ± 0.92	**95.79 ± 0.60**	97.38 ± 0.34	83.44 ± 0.72	51.78 ± 1.95	70.59 ± 1.15	84.76 ± 0.92	91.52 ± 0.68	95.89 ± 0.40
SE	90.60 ± 0.55	89.20 ± 0.72	**89.43 ± 0.80**	95.59 ± 0.48	84.83 ± 0.80	93.85 ± 0.68	97.63 ± 0.38	91.59 ± 0.72	45.64 ± 1.92	62.12 ± 1.15	84.11 ± 0.92	91.00 ± 0.85	96.05 ± 0.55
RSA	**97.31 ± 0.64**	93.81 ± 0.68	86.47 ± 1.42	92.17 ± 1.16	88.30 ± 1.44	85.43 ± 1.59	96.39 ± 0.58	97.80 ± 0.44	**70.03 ± 1.95**	**72.58 ± 1.72**	**88.23 ± 1.39**	91.77 ± 1.58	95.70 ± 0.66

Bold values indicate the optimal values.

**Table 8:** Results of the RSA kernel size comparison experiment.

Kernel Size	*IoU*										*mIoU*	*mA*	*OA*
	Highline	Lowline	Groundline	Tower	Ground	Road	House	Tree	Obatacle	Car			
1 × 1	92.42 ± 0.85	88.57 ± 0.92	91.47 ± 0.85	96.05 ± 0.87	91.18 ± 1.01	**94.99 ± 0.73**	97.94 ± 0.31	86.00 ± 1.72	56.05 ± 1.95	55.31 ± 2.45	86.75 ± 1.56	91.25 ± 0.89	97.06 ± 0.45
3 × 3 (ours)	**96.47 ± 0.38**	**96.16 ± 0.38**	**93.75 ± 0.50**	93.08 ± 0.56	**92.16 ± 0.68**	93.24 ± 0.52	97.39 ± 0.46	**98.08 ± 0.34**	67.54 ± 1.24	**79.17 ± 1.31**	**90.05 ± 0.82**	**94.18 ± 0.56**	97.11 ± 0.48
5 × 5	91.24 ± 0.85	90.07 ± 0.92	90.03 ± 1.15	**96.81 ± 0.98**	86.37 ± 1.45	97.14 ± 1.23	97.94 ± 0.41	86.10 ± 1.02	64.32 ± 1.95	74.07 ± 2.05	86.75 ± 1.46	92.20 ± 0.89	**97.34 ± 0.45**
7 × 7	86.23 ± 0.92	94.26 ± 0.65	82.26 ± 1.16	96.56 ± 0.63	85.12 ± 1.72	94.28 ± 1.45	**98.04 ± 0.45**	85.17 ± 1.67	**70.40 ± 2.12**	65.41 ± 2.34	85.47 ± 1.72	90.72 ± 0.78	95.10 ± 0.89

Bold values indicate the optimal values.

## Data Availability

The data presented in this study are available on request from the author. because the data are part of an ongoing study and there are privacy restrictions. Requests to access the datasets should be directed to 2024282070146@whu.edu.cn.
